# Homozygous Pro1066Arg *MYBPC3* Pathogenic Variant in a 26Mb Region of Homozygosity Associated with Severe Hypertrophic Cardiomyopathy in a Patient of an Apparent Non-Consanguineous Family

**DOI:** 10.3390/life12071035

**Published:** 2022-07-12

**Authors:** Raquel Rodríguez-López, Javier García-Planells, Marina Martínez-Matilla, Cristian Pérez-García, Amor García Banacloy, Carola Guzmán Luján, Otilia Zomeño Alcalá, Joaquina Belchi Navarro, Juan Martínez-León, Rafael Salguero-Bodes

**Affiliations:** 1 Laboratory of Molecular Genetics, Clinical Analysis Service, Consortium General University Hospital, 46014 Valencia, Spain; guzman_reme@gva.es (C.G.L.); zomenyo_oti@gva.es (O.Z.A.); 2 Igenomix, 46980 Valencia, Spain; javier.garcia@igenomix.com (J.G.-P.); marina.martinez@igenomix.com (M.M.-M.); cristian.perez@igenomix.com (C.P.-G.); 3 Department of Surgery, Faculty of Medicine, University of Valencia, 46010 Valencia, Spain; amor.garcia@uv.es; 4 Cardiology Service, Consortium General University Hospital, 46014 Valencia, Spain; belchi_qui@gva.es; 5 Cardiovascular Surgery Service Hospital La Fe, 46026 Valencia, Spain; juan.martinez-leon@uv.es; 6 Cardiology Department and Research Institute Hospital Universitario, Hospital Universitario 12 de Octubre, 28041 Madrid, Spain; rafael.salguero@salud.madrid.org; 7 Centro de Investigación Biomédica en Red de Enfermedades Cardiovasculares (CIBERCV), Carlos III Health Institute, 28029 Madrid, Spain; 8 Medicine Department, Faculty of Medicine, Complutense University of Madrid, 28040 Madrid, Spain

**Keywords:** *MYBPC3*, region of homozygosity, *MYH7*, cardiac phenotype, HPO terms

## Abstract

*MYPBC3* and *MYH7* are the most frequently mutated genes in patients with hereditary HCM. Homozygous and compound heterozygous genotypes generate the most severe phenotypes. A 35-year-old woman who was a homozygous carrier of the p.(Pro1066Arg) variant in the *MYBPC3* gene, developed HCM phenocopy associated with left ventricular noncompaction and various degrees of conduction disease. Her father, a double heterozygote for this variant in *MYBPC3* combined with the variant p.(Gly1931Cys) in the *MYH7* gene, was affected by HCM. The variant in *MYBPC3* in the heterozygosis-produced phenotype was neither in the mother nor in her only sister. Familial segregation analysis showed that the homozygous genotype p.(Pro1066Arg) was located in a region of 26 Mb loss of heterozygosity due to some consanguinity in the parents. These findings describe the pathogenicity of this variant, supporting the hypothesis of cumulative variants in cardiomyopathies, as well as the modulatory effect of the phenotype by other genes such as *MYH7.* Advancing HPO phenotyping promoted by the Human Phenotype Ontology, the gene–disease correlation, and vice versa, is evidence for the phenotypic heterogeneity of familial heart disease. The progressive establishment of phenotypic characteristics over time also complicates the clinical description.

## 1. Introduction

Hypertrophic cardiomyopathy (HCM) is defined by the presence of increased left ventricular wall thickness that cannot be explained solely by abnormal loading conditions.

HCM is the most commonly known cardiovascular genetic disorder with an estimated prevalence of 1/500 [1]. The clinical heterogeneity of this genetic disorder has a very high consequence of genetic and environmental factors, ranging from asymptomatic patients to sudden cardiac death as the first sign. HCM is characterized by incomplete penetrance and variable expressivity, even in cases with one identified pathogenic variant in the context of the same family. Pathogenic variants in genes coding for sarcomeric proteins represent the most common genetic subtype of HCM [2]. Specifically, *MYPBC3* is the most frequently mutated gene in patients with inherited hypertrophic cardiomyopathy (HCM), representing 40–50% of all HCM pathogenic variants [3], followed by *MYH7*, which is responsible for approximately 30% of HCM pathogenic variants.

HCM is inherited following an autosomal dominant pattern of inheritance; nevertheless, multiple cases with homozygous, double heterozygous or compound heterozygous pathogenic variants are frequent (5–10%) and used to be associated with a more severe phenotype [4,5,6] and early onset HCM [7]. Moreover, the type of pathogenic variants also causes important repercussions in the phenotype; for example, bi-allelic truncating *MYBPC3* mutations that are associated with neonatal cardiomyopathy lead to heart failure and death within the first year of life [8,9,10].

Herein we present a family case with two patients, father and daughter, with different clinical manifestation associated with different mutational events. The father had a double heterozygous mutation for a novel variant of uncertain significance (VUS) in the *MYH7* and the *MYBPC3* genes. The daughter was identified as homozygous carrier of the variant in the *MYBPC3* gene, identified in the father. Although consanguinity was not reported, a 26 Mb region of homozygosity in the genomic region encompassing the *MYPC3* gene generated the homozygous condition.

Elucidating the phenotype–genotype correlations of these complex cases allows a more rigorous approximation of the deleteriousness of each identified genotype by combining the two familiar segregating amino acid changes. Comprehensive phenotypic description of HPO facilitates prediction of clinical course and prognosis of similarly affected patients and families who carry these VUSs in the *MYH7* and *MYBPC3* genes. Enhancing knowledge of these disorders ensures that individualized and scientifically argued clinical management becomes a reality, providing more accurate clinical risk stratification and better genetic counseling to the families.

## 2. Materials and Methods

### 2.1. Patients/Clinical Description

The proband of the family was referred to the Familial Heart Disease Consultation of our hospital, cataloged as one of the Reference Consultations in Hereditary Heart Diseases and Vasculopathies among 13 Centers, Services and Reference Units of the National Health System (CSUR-SNS) dedicated to this specific healthcare process (https://www.sanidad.gob.es/profesionales/CentrosDeReferencia/CentrosCSUR.htm (accessed on 20 May 2022); coordination assigned to the corresponding author. The patient was performing a light physical warm-up prior to starting physical activity when he presented a sudden loss of consciousness, and was then treated with a semi-automatic defibrillator. Ventricular fibrillation was detected, with symptoms of cardiogenic shock. In the nearest medical center, he received advanced cardiopulmonary recovery maneuvers, and administration of up to 4 doses of adrenaline and 300 mg of amiodarone; recovery was achieved. The family reported 2 previous isolated syncopes with complete recovery. He did not previously show other symptoms of heart failure and/or palpitations. Coronary angiography was performed without finding epicardial artery lesions. Conventional magnetic resonance imaging (MRI) confirmed the existence of hypertrophic cardiomyopathy, and the patient’s severe increase in the thickness of the heart muscle and the patient’s size and personal history led to the decision to implant a defibrillator.

All first-degree relatives of the patient were evaluated in the Familial Heart Disease Consultation, according to the protocol agreed by the CSUR of Hereditary Heart Diseases and Vascular Diseases. The patient has two daughters, one of whom was diagnosed with apical hypertrophic cardiomyopathy at the age of 26. Due to the genetic findings, the mother of his two daughters was also genetically evaluated and studied.

The family history of both branches of the family was extensively investigated, going back three generations. A family segregation study of both identified genetic variants was carried out. In the family of the proband (I:1), both variants were analyzed in his two siblings, a 60-year-old man and a 55-year-old woman. In the mother’s family (I:2), the MYBPC3 gene variant was analyzed in her mother (89 years old) and her two siblings, a 57-year-old man and a 48-year-old woman.

### 2.2. Genetic Analysis

Whole exome sequencing (WES) trio analysis (affected 26-year-old woman and both parents) was performed on DNA extracted from individuals. WES libraries were constructed using the Nextera DNA Flex Pre-Enrichment Library Prep and Illumina Exome Panel and sequenced with S2/S4 Reagent Kits (Illumina) on the NovaSeq 6000 Sequencing System (Illumina). Raw sequencing data were processed by an in-house bioinformatics pipeline (v1.0). The reads were mapped to the human genome reference (GRCh37/hg19) and duplicated reads were marked before variant calling and annotation. The ExomeDepth tool was used [11] for the detection of germline copy-number variations (CNVs). A minimum of a 20× reading depth was obtained for 95% of the coding regions (±10 nt). Genetic variants were filtered and prioritized according to a phenotype-driven gene panel manually selected and curated for 208 genes associated with cardiomyopathies (Supplementary Table S1, allele frequency and clinical evidence. The clinical classification of the genetic variants in those genes was carried out according to the recommendations of the American College of Medical Genetics (ACMG) [12] and Association for Clinical Genomic Science [13].

The methodology followed for the detection of runs of homozygosity (ROHs) makes use of detected sample variants, evaluating the distribution of the variant allele frequency along virtually generated regions on the genome. Quality and depth filters of 150 and 30× were used to remove those variants in inconclusive or difficult-to-call regions of the genome, followed by the elimination of the variants in the X and Y chromosomes. Finally, only variants between a variant allele fraction of 0.4 and 0.6 or over 0.9 were used. The in-house-developed algorithm built a series of windows through the entire genome, each of them containing 35 contiguous SNPs. Each window was evaluated separately to assess the heterozygosity of the region, checking if a ROH had occurred. Contiguous windows were aggregated in a final step to obtain an ending result.

Family segregation analysis of both variants in the other five members of the family was performed by Sanger sequencing on an Applied Biosystems 3130 sequencer.

## 3. Results

### 3.1. Clinical Evaluation

All family members underwent an exhaustive clinical evaluation, according to the protocol agreed by the CSUR of Hereditary Heart and Vascular Diseases.

The results of the clinical evaluation carried out on the father revealed: Cardiac MRI: Normal left ventricular (LV) volumes and both segmental and global systolic function (left ventricular ejection fraction LVEF: 67%) with hypertrophy of the mid and apical segments of the left ventricle (131 g/m^2^), and with a maximum wall thickness of 30 mm in the apical septum. Left ventricular outflow tract (LVOT) obstruction was not detected. Right ventricular morphology, volume and systolic function (right ventricular ejection fraction RVEF: 60%) were normal. Normal atria. Ascending and descending aorta of normal caliber and morphology. Normal first-pass infusion at rest of 0.075 mmol/kg intravenous gadobenate dimeglumine. Late gadolinium enhancement (LGE) showed a severe pattern of focal fibrosis in the inferior and anterior septum and a diffuse pattern in apical segments. Ergometry: No notable findings. Twenty-four-hour Holter ECG: Sinus rhythm throughout the recording with short bursts of nonsustained ventricular tachycardia (NSVT). During follow-up, the last echocardiographic study performed at the age of 60 concluded: Left ventricle had normal volumes and severe apical hypertrophy, and preserved global systolic function and a moderate midventricular gradient (instantaneous peak gradient of 38 mmHg). No valve disease. Mild dilatation of the left atrium. Right ventricle had normal diameters with severe apical hypertrophy and preserved systolic function. Mild tricuspid regurgitation, which estimated approximate systolic pulmonary artery pressure (PSP) of 35 mmHg.

The patient has two daughters, one of whom was diagnosed with apical hypertrophic cardiomyopathy at the age of 26 years. Cardiac MRI: Left ventricle had increased mild volumes with predominantly mid-septal hypertrophy with marked trabeculation of the mid-apical free wall, fulfilling criteria of noncompaction; LGE suggestive of fibrosis in the septum. Nondilated RV with normal systolic function. LV with concentric hypertrophy (interventricular septum 21 mm, posterior wall 25 mm) without LVOT obstruction. Ablation of the slow pathway was performed, and was effective and without complications. Holter ECG: Sinus rhythm throughout the recording with a mean heart rate (HR) of 58 bpm. Low-density supraventricular and ventricular extrasystole (1% beats), monomorphic (upper axis—hyper-right, negative in V3 and V4, suggesting LV anteroapical origin); five doublets and no episodes of NSVT.

It was decided to implant an ICD and treat with bisoprolol, 2.5 mg daily.

**Figure 1:**
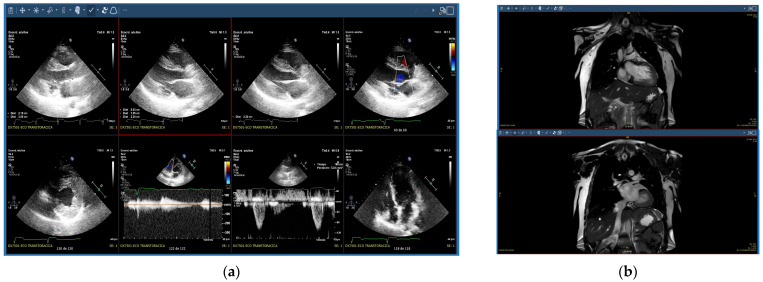
(**a**) Echocardiographic study in the affected daughter, (**b**) magnetic resonance imaging report of the affected daughter.

**Figure 2:**
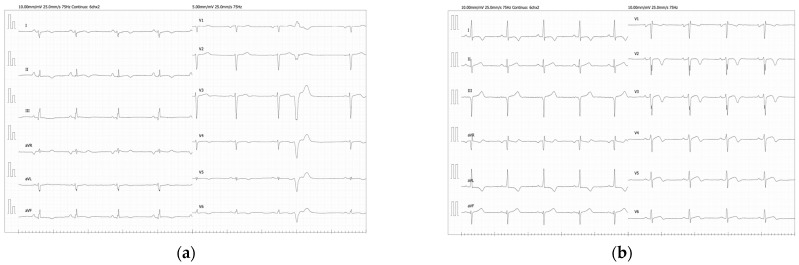
Electrocardiographic study of the daughter (**a**) and her father (**b**) at the ages of 36 and 60 years, respectively. Abnormal ECGs: (**a**) Sinus rhythm with occasional premature ventricular complexes. T-wave abnormality, possible inferior ischemia, possible right ventricular hypertrophy, left atrial enlargement. (**b**) Sinus rhythm, RSR (OR) in lead V1/V2, coincident with ventricular conduction delay. Left ventricular hypertrophy with abnormal repolarization. Possible left atrial enlargement. Moderate left-axis deviation.

In follow-up, the last clinical evaluation carried out when the daughter was 35 years of age showed: Cardiac MRI: Nondilated left ventricle without global hypertrophy (LVMI: 62 g/m^2^) but with asymmetric wall thickness, and aneurysmal segments such as the apex and basal-lateral segment. The maximum diastolic thickness was 20 mm on the mid-lateral segment. Normal systolic function and LVEF of 61%. Nondilated right ventricle with normal systolic function and RVEF of 50%. Dilated left atrium (T1 increased up to 132 ms, T2 increased up to 70 ms). No edema was detected on T2-weighted sequences. Normal perfusion sequences. An extensive area of epicardial LGE with high transmurality was observed in all segments, except in those with greater hypertrophy, which were relatively spared (Figure 1). Echocardiographic study: LV hypertrophy with mid-septal predominance (up to 19–20 mm) without LVOT obstruction. LV with normal volume with preserved systolic function (LVEF 60–65%) and presence of apical hypokinesia. Left atrium had mild dilatation. Nondilated right ventricle, with normal systolic function. Mild tricuspid regurgitation, estimating PSP of 25 mmHg. Twenty-four-hour Holter ECG: Sinus rhythm throughout the recording, with a mean HR of 71 bpm. Low-density supraventricular and ventricular extrasystole (1%) with two morphologies, with a single NSVT of 20 beats at initial HR of 125–130 bpm and progressive deceleration up to 90 bpm with the same morphology as one of the ventricular extrasystole (Figure 2).

After the complete clinical evaluation according to agreed protocols, the exhaustive phenotypic description of the father and his affected daughter, according to HPO terms, is detailed in Table 1. The genotype–phenotype correlation evidenced only 6 coincident terms (highlighted in grey), among 16 and 13 defining characteristics of the father and daughter, respectively.

Clinical study on the other daughter and mother (including electrocardiogram and echocardiographic study) showed no abnormalities.

### 3.2. Genetic Variant Analysis

**Figure 3:**
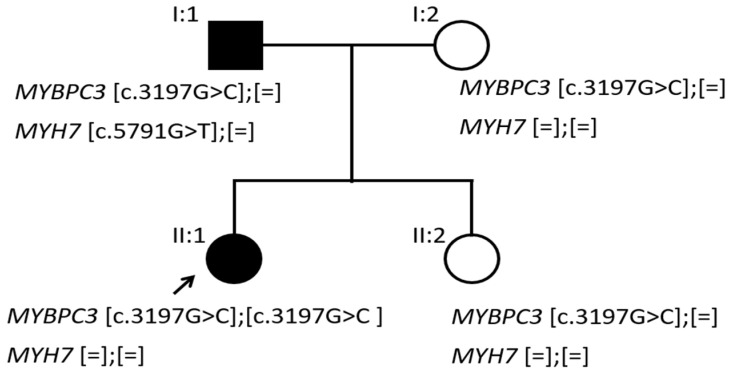
Family tree showing the segregation analysis of the two potential deleterious variants identified.

**Figure 4:**
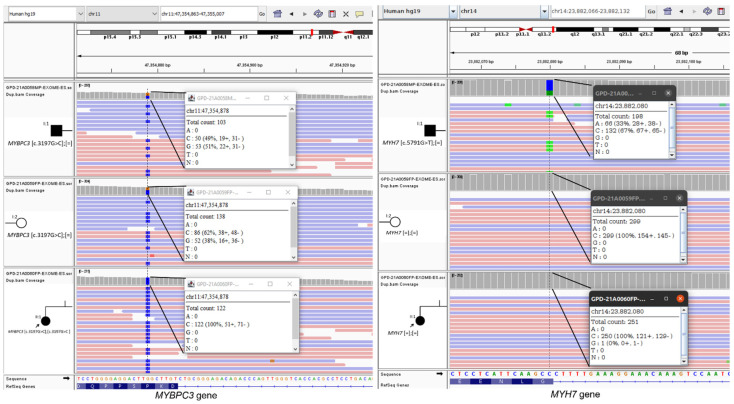
Visualization of the two identified variants, MYBPC3 (**left side**) and MYH7 (**right side**) by using the Integrative Genomics Viewer (IGV; https://software.broadinstitute.org/software/igv/ (accessed on 20 May 2022)). Samples are indicated at the left side of each picture. As a measure of variant quality, the number of reads and percentage of each nucleotide are shown in a window for each case.

Molecular analysis showed that the father was double heterozygous for a novel VUS in the *MYH7* gene (NM_000257.2 (MYH7): c.5791G>T; p.(Gly1931Cys)) and for a VUS in the *MYBPC3* gene (NM_000256.3 (MYBPC3): c.3197C>G; p.(Pro1066Arg)). The family segregation study (Figure 3) revealed that the affected daughter (II:1) was homozygous for the variant in the *MYBPC3* gene c.(3197C>G); (3197C>G), whereas the mother (I:2) and the unaffected daughter (II:2) were heterozygous for the same rare missense in the *MYBPC3* gene (Figure 4). It was found that the father had inherited each of the variants he carried from each of his parents.

The novel variant in the *MYH7* gene (c.5791G>T; p.(Gly1931Cys)) was detected in the affected father (I:1). This change was not detected in population databases (more than 125,000 exomes of the gnomAD database). Genomic position was conserved and in silico prediction was inconclusive regarding the impact of this variant on protein structure and function. The variant p.(Gly1931Cys) is located in exon 40 of the *MYH7* gene, which is outside the head domain (amino acids 181–937, NM_000257), where most of the pathogenic variants in this gene have been described [14]. For these reasons, this variant was classified as a variant of uncertain significance.

The variant (c.3197C>G; p.(Pro1066Arg)) in exon 29 of the *MYBPC3* gene (NM_000256.3) was previously classified as an uncertain significance variant by two submitters in the ClinVar database (ClinVar variation ID: 1171602). This change has not been reported in gnomAD; however, it was recently reported in an individual with restrictive cardiomyopathy, who also carried a pathogenic variant in the *MYH7* gene (c.2302G>A; p.(Gly768Arg)), which was also in his unaffected father [15]. The majority of in silico pathogenicity prediction tools support a deleterious effect on the gene and genomic position is conserved. This variant was firstly classified as a variant of uncertain significance due to lack of evidence supporting its pathogenicity. However, after the family co-segregation, it was reclassified as likely pathogenic.

Other rare variants (MAF < 0.001) in the 208 analyzed genes after filtering and prioritizing analysis are described in Supplementary Table S2.

The family history of the proband (I:1) revealed that his father had died at the age of 41 from a poorly defined pulmonary pathology and his mother at the age of 72 due to a colon neoplasm. The two brothers were alive and healthy. No other family history related to cardiac pathologies, arrhythmic events and/or sudden death was reported. The study of the genetic variants identified in the proband in both brothers ruled out that they were carriers of any of them. In the family of the mother of the daughters (I:2), the maternal grandmother was 89 years old and healthy, and both brothers of the mother were alive and healthy as well. Her father had died at the age of 79 from a lung neoplasm. The only reference to a possible relationship was that of a paternal uncle who died of acute myocardial infarction at 69 years of age; his sons lived without incident. It was found that neither the mother nor the mother’s siblings (I:2) were carriers of the analyzed variant in the *MYBPC3* gene.

### 3.3. Homozygosity Analysis

**Figure 5:**
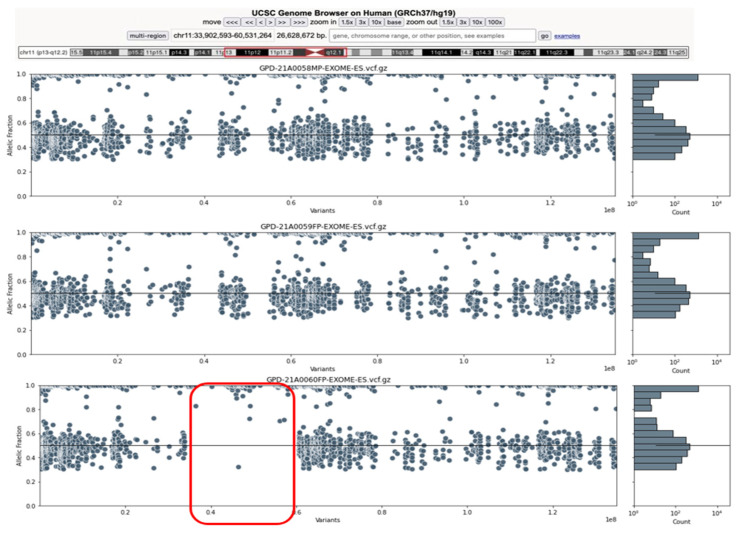
The images describe the distribution of the variants of the exome trio analysis, the order being father, mother and patient. The left plot visualizes the variant distribution on chromosome 11, pointing out the detected ROH event on the 11:33902593–60531264 region (red frame). On the vertical axis the variant frequency on each sample is identified, whereas the horizontal axis describes the position in the GRCh37 reference genome. On the right, a histogram describing the distribution of the variants in the region is shown.

Although initially there was no consanguinity reported in the family, the presence of homozygosis in a low-frequency variant in the population, the high quantity of homozygous variants in the same region and the absence of CNVs in the *MYBCP3* gene in the affected daughter (II:1) brought us to the conclusion of a consanguineous family. The evaluation for ROHs by the application of an in-house algorithm on the patient allowed us to discover a 26 Mb ROH ranging from 11:33902593–60531264 (referring to the GRCh37 reference genome) (Figure 5).

To support the potential consanguinity gathered from the homozygosity analysis, additional variants including homozygosis in the daughter, heterozygosis in both fathers and a low frequency in the population (gnomAD) were investigated inside the ROH.

Four rare (MAF < 0.01) homozygous variants detected inside the ROH in the affected daughter were also present in the heterozygous state in both parents (Table 2), which would support the potential consanguinity.

No other large regions (greater than 5 Mb) of runs of homozygosity (ROHs) were identified in the affected daughter (II:1), nor did the global percentage of ROHs estimated by WES data add up to more than 3%. The number and size of runs of homozygosity (ROHs) provide an estimation of the inbreeding relationship between any two individuals. ROHs longer than 5 Mb suggest the presence of a shared maternal and paternal ancestor during the last six generations [16]. None of these large blocks of loss of heterozygosity were identified in the patient’s unaffected sister (II:2). In order to adequately complete the genetic counseling in patient II:1 and her family, all the genes located in the 26 Mb chromosomal region inside the ROH (11:33902593–60531264) were exhaustively evaluated. The existence of variants of possible pathogenicity in any gene associated with an autosomal recessive pattern and/or X-linked inheritance was ruled out.

## 4. Discussion

Myosin-binding protein C (MYBPC3) and myosin heavy chain-β (MYH7) are proteins involved in the thick filament of the sarcomere [17]. These proteins are codified by *MYBPC3* and *MYH7* genes and pathogenic variants on those genes are responsible for approximately three-quarters of the identified deleterious changes in patients with hypertrophic cardiomyopathies (HCMs) [18].

Some studies suggest a more severe phenotype and an earlier disease onset in patients carrying pathogenic variants in *MYH7* compared to *MYBPC3*, although evidence is scarce [19]. In the same way, patients with two deleterious mutations in the *MYBPC3* gene had severe diseases and a trend toward a younger age at presentation when compared with patients with a single causal variant [20]. Approximately 5% of the HCM family cases present complex genetic results with more than one causal mutation in sarcomere genes: homozygotes for the same pathogenic variant, compound heterozygotes (two different deleterious changes in the same gene) or double heterozygotes (two different pathogenic variants in different genes) [18,19].

In this study we reported a family case of two affected members with HCM, with different clinical features and different genotypes. The father, 59 years old with severe biventricular apical hypertrophy, was double heterozygous for a novel variant of uncertain significance (VUS) in the *MYH7* gene (c.5791G>T; p.(Gly1931Cys)) and for a VUS in the *MYBPC3* gene (c.3197C>G; p.(Pro1066Arg)). The daughter, 35 years old with apical HCM, left ventricular noncompaction and septal fibrosis, was identified as a homozygous carrier of the variant in the *MYBPC3* gene c.(3197C>G); (3197C>G) was identified in the father. Although consanguinity was not reported, the unaffected mother was also heterozygous for the *MYBPC3* variant, and the daughter showed a 26 Mb region of homozygosity in the genomic region encompassing the *MYPC3* gene where the homozygous variant is located. In addition, her only sister was also a heterozygous carrier of the p.(Pro1066Arg) of *MYBPC3* and did not present any signs or symptoms of associated heart disease.

Thus, there was a 35-year-old woman with HCM and noncompaction of the left-ventricle, who was a carrier of the homozygous variant p.(Pro1066Arg) *MYBPC3* included in a homozygous region of 26 Mb. This variant was also detected in a heterozygous state in her mother and her only sister, both without associated phenotypic characteristics. Her father, who was affected by HCM, also carried the heterozygous variant p.(Gly1931Cys) in the *MYH7* gene. The variant p.(Pro1066Arg) was previously detected in heterozygosity in healthy individuals, but was not previously described in homozygosis in any patient or individual. Other homozygous mutations in the *MYBPC3* gene were described, hypothesizing a double-dose effect [18]. Double heterozygotes for mutations in the *MYH7* and *MYBPC3* genes were also described, and exhaustive phenotypic studies were also carried out, trying to compare the agreed echocardiographic parameters to describe the HCM associated with each of the genes [19].

### 4.1. Genotype-Phenotype Correlation

Although consanguinity was not reported in this family, the affected father (I:1) and two unaffected members (mother, I:2, and sister, II:2) were heterozygous carriers for the rare missense variant in the *MYBPC3* gene. The affected daughter (II:1), however, was homozygous for this variant. These findings strengthen the pathogenicity of this variant and suggest that, although this change detected in isolation may lead to a subtle phenotype, it could also act as a genetic modifier in the presence of another pathogenic variant (as could be the case of the affected father (I:1), with a double heterozygote of another missense variant in the *MYH7* gene), or even as a pathogenic variant when it is present in both alleles of the patient, as in the case of the affected daughter (II:1).

Both patients have severe phenotypes of HCM. The father (I:1) has biventricular apical hypertrophy and suffered a cardiorespiratory arrest (HP:0031992, HP:0006543, HP:0001663). The affected daughter (II:1) has HCM with a high arrhythmogenic component (HP:0031992, HP:0004763, HP:0001663, HP:0006682), requiring cardiac ablation at age 27; she also has marked mid-apical trabeculation, meeting the criteria for noncompaction (HP:0031195, HP:0030682). These results support a cumulative variant hypothesis in hypertrophic cardiomyopathy.

These findings support the hypothesis of the accumulation/combination of variants of a certain functional capacity, both monogenic and polygenic, to generate the existing phenotypic diversity both in HCM cases and in other familial heart diseases. The p.(Pro1066Arg) genotype in homozygosis in the *MYBPC3* gene in the symptomatic female patient from the age of 26 showed its ability to produce a phenocopy of the pathologies that occur with HCM associated with various degrees of conduction disease, similar to the causality of pathogenic variants in the *PRKAG2* or *LAMP2* genes (which can cause various degrees of conduction disease and cardiac hypertrophy that mimic HCM) or mitochondrial pathogenic variants that cause MELAS syndrome (hypertrophic cardiomyopathy also associated with conduction disorders) [21].

### 4.2. Homozygosity Analysis

Homozygosity mapping is a classic approach to encircle potential genomic regions where a disease-causing recessive variant might be present. The detection of homozygous genomic regions would limit the number of loci potentially involved in the etiology of recessive disorders.

The sensitivity of WES for detecting runs of homozygosity (ROHs) is lower than classical approaches, such as those through genome-wide single-nucleotide variant arrays (GWAS), but the WES approach yields very precise and reliable estimates of homozygosity rates using homozygosity series with a 1000 kb window, which is especially higher in coding regions [22].

The exome is not the best way to perform homozygosity analysis, as it is not representative and provides information for only 1% of the genome. However, if we extrapolate with the available information of the presence of a homozygosity region of 26 Mb compared to the 3050 Mb of the genome, we can calculate that the parents share at least 0.78% of the genome, which suggests some consanguinity or belonging to a population with some inbreeding. In no case were we able to conclude that the patient or the family had a higher degree of consanguinity than those considered usual population indices in our setting. Neither did the patient or her only sister have a greater susceptibility for recessive genetic diseases. Only the existence of a large linkage disequilibrium block flanking the *MYBPC3* gene (chromosomal region 11:33902593–60531264) was demonstrated, which was maintained in individuals from the very close geographic range of both parents (populations of moderate/small population size in the geography of the Valencian Community, close to Valencia) and which the patient II:1 had inherited in homozygosity.

## 5. Conclusions

The heterozygous genotype of the variant considered to be of uncertain significance c.3197C>G; p.(Pro1066Arg) in the *MYBPC3* gene did not show sufficient deleterious capacity without generating clinical manifestations in carrier individuals of the family with ages even beyond the fifth decade of life.

However, the genotype of this variant in the *MYBPC3* gene in homozygosis strongly suggests serious clinical implications manifested from the second decade of life. Previous evidence of similar high-risk and penetrance genotypes, some located in the same or very close/similar functional domains of the Mybpc3 protein, have been described [7,20,23,24,25,26].

Evidence of the cumulative theory of certain amino acid change variants in heart disease susceptibility genes was shown for the heterozygous genotype of *MYBPC3,* to be indeed pathogenic when the heterozygous genotype of the c.2302G>A; p.(Gly768Arg) variant in the *MYH7* gene was combined. The expressed phenotype, as well as its form and age of presentation, was different. The literature also exposes very similar cases of this combination [14,18,19].

The fact of having carried out a complete clinical exome study on the four members of the family ruled out the existence of other genetic variants that could be acting as modifier genes of the phenotypes, both risk and protective. No other genetic changes were observed whose molecular characteristics and/or low population frequencies deserved consideration in this case.

Although the coincident HPO terms in the father and his affected daughter corroborate the capacity and similar genetic determination of both genes involved in HCM and sudden cardiac death, it can be concluded that (at least in this case) the *MYH7* gene carries a more structural change and the *MYBPC3* gene generates greater alteration of the cardiac conduction system. Without being able to speak of haploinsufficiency associated with the *MYBPC3* gene variant, it is evident that its mild/moderate functional capacity in heterozygosity reaches a much more serious repercussion when both alleles are affected.

We consider it is essential to promote the phenotypic description of patients according to the HPO terminology to facilitate the digital treatment of clinical data and, with it, the implementation of algorithmic medicine in our health systems. Data mining-based tools aim to assist clinicians in their diagnoses and clinical decisions in the field of hereditary heart disease; the generalizability and explanatory power of established consensus algorithms should be better assessed [27].

The main limitation to conclude our manuscript was not having been able to include a functional study that reliably demonstrates that the genotype–phenotype correlations indicated in the affected individuals (affected father and daughter) were the only and sufficient molecular cause. Functional studies, such as a mouse model carrier of the different family genotypes identified, would be able to demonstrate that they are sufficient to cause heart disease in carrier animals, combined and/or even isolated. To our knowledge, the mouse model is the functional assay most capable of elucidating the mechanisms of heart disease caused by a specific genetic variant.

## Figures and Tables

**Table 1:** Clinical phenotypic description of the father and his affected daughter, according to HPO terms.

Father’s HPO Terms	Daughter’s HPO Terms
Structural findings	
Apical hypertrophic cardiomyopathy HP:0031992	Apical hypertrophic cardiomyopathy HP:0031992
Myocardial fibrosis HP:0001685	Myocardial fibrosis HP:0001685
Concentric hypertrophic cardiomyopathy HP:0005157	Concentric hypertrophic cardiomyopathy HP:0005157
Left atrial enlargement HP:0031295	Left atrial enlargement HP:0031295
Right ventricular hypertrophy HP:0001667	Right ventricular hypertrophy HP:0001667
Interstitial cardiac fibrosis HP:0031329	Asymmetric septal hypertrophy HP:0001670
	Left ventricular hypertrophy HP:0001712
	Ventricular septal hypertrophy HP:0005144
	Apical hypertrabeculation of the left ventricle HP:0031195
	Left ventricular noncompaction HP:0030682
Arrhythmogenic findings	
Abnormal T-wave HP:0005135	Abnormal T-wave HP:0005135
Ventricular tachycardia (TVNS) HP:0004756	Premature ventricular contraction HP:0006682
Syncope HP:0001279	Paroxysmal supraventricular tachycardia (PSVT) HP:0004763
Ventricular fibrillation (VF) HP:0001663	
Cardiorespiratory arrest (PCR) HP:0006543	
Aborted sudden cardiac death HP:0031628	
Abnormal QRS voltage HP:0025076	
Supraventricular tachycardia HP:0004755	
Atrial fibrillation HP:0005110	
Cardiac conduction abnormality HP:0031546	

**Table 2:** Rare homozygous variants detected inside the ROH in daughter, present in heterozygous state in both parents.

Chromosomal Position	gnomAD Frequency (MAF) *	Daughter (II:1)	Father (I:1)	Mother (I:2)
chr11:34153131G>A	0.0003842	Hom	Het	Het
chr11:55944242A>T	0.002398	Hom	Het	Het
chr11:58125873G>A	0.0002524	Hom	Het	Het
chr11:59245301G>A	0.0001844	Hom	Het	Het

* MAF: minor allele frequency; Hom: homozygous; Het: heterozygous.

## Data Availability

Not applicable.

## Supplementary Materials

The following supporting information can be downloaded at: https://www.mdpi.com/article/10.3390/life12071035/s1, Supplementary Table S1 and Supplementary Table S2.
